# Constitutive overexpression of a novel 21 kDa protein by Hodgkin Lymphoma and Aggressive Non-Hodgkin Lymphomas

**DOI:** 10.1186/1476-4598-7-12

**Published:** 2008-01-24

**Authors:** Minglong Zhou, Faisal M Fadlelmola, Jason B Cohn, Brian Skinnider, Randy D Gascoyne, Diponkar Banerjee

**Affiliations:** 1 Centre for Translational and Applied Genomics (CTAG), Department of Pathology and Laboratory Medicine, British Columbia Cancer Agency, Vancouver Cancer Centre, BC V5Z 4E6, Canada; 2 Samuel Lunenfeld Research Institute, Toronto, Canada; 3 University of British Columbia, Vancouver, British Columbia, Canada

## Abstract

**Background:**

CD30, a 120 kDa surface phosphorylated protein is a member of tumour necrosis/nerve growth factor receptor (TNF/NGFR) family and constitutively expressed by Hodgkin and Reed-Sternberg (HRS) cells of Hodgkin lymphoma (HL) and the neoplastic cells of Anaplastic Large Cell Lymphoma (ALCL). A disease-specific protein marker is yet to be identified in Hodgkin lymphoma cells. In order to define HL-specific biomarkers, novel murine monoclonal antibodies were developed in our laboratory.

**Results:**

Murine monoclonal antibodies (mabs) were raised against the B3 sub clone of HL-derived cell line KM-H2. Two of these mabs (clone R23.1 mab and clone R24.1 mab) are IgG_1 _class antibodies that recognize a 21 kDa protein present at the cell membrane and in the cytoplasm in HL-derived cell lines. Clone R24.1 mab recognizes a formalin-resistant epitope and labels HRS cells in tissue samples from patients with HL of the classical type, ALCL, and subsets of T and B cell aggressive Non-Hodgkin Lymphomas (NHL). The antigen recognized by the clone R23.1 mab and clone R24.1 mab does not share epitopes with CD30 cluster regions A, B, or C, and, unlike CD30, is not expressed by phytohemagglutinin (PHA) activated T cells.

**Conclusion:**

The 21 kDa protein detected by clone R23.1 and clone R24.1 mabs is a novel membrane-associated protein that may be a potential marker for the diagnosis and targeted therapy of HL and aggressive T and B cell NHL.

## Background

Hodgkin and Reed-Sternberg (HRS) cells of HL and the neoplastic cells of Anaplastic Large Cell Lymphoma (ALCL) constitutively express CD30 [1]. CD30 has been characterized as a 120 kDa surface phosphorylated glycoprotein, and is a member of the tumour necrosis factor/nerve growth factor receptor (TNF/NGFR) family [2]. Currently available antibodies against CD30 recognize one of three clusters designated as A, B, and C. For instance, antibodies Ki-2, Ki-4, Ki-5, Ki-7, Ber-H2, HRS-1 and HRS-4 recognize cluster A, antibodies Ki-1, Ki-6, and M67 recognize cluster B, and antibodies Ki-3, M44, HeFi-1 and C10 recognize cluster C[3].

CD30, however, does not have disease-specificity, as it is an activation-associated antigen. It is expressed by activated T and B cells, HTLV-I or HTLV-II transformed T cells, EBV-transformed B cells [4], ALCL [5], mediastinal diffuse large B cell lymphoma [6], other diffuse large B cell lymphomas [7], follicular centre cell lymphoma [8], and testicular embryonal carcinoma cells [9].

The identification of cell surface molecules that are not activation-associated markers, and have specificity for HRS cells thus remains a desirable goal. To this end, we have developed and characterized 2 novel monoclonal antibodies, R23.1 and R24.1, that recognize a 21 kDa molecule expressed by H/RS and ALCL cells, but not by phytohemagglutinin (PHA) activated CD30+ T lymphocytes.

## Results

### Reactivity of R23.1 and R24.1 against CD30+ and CD30- cell lines

**Figure 1:**
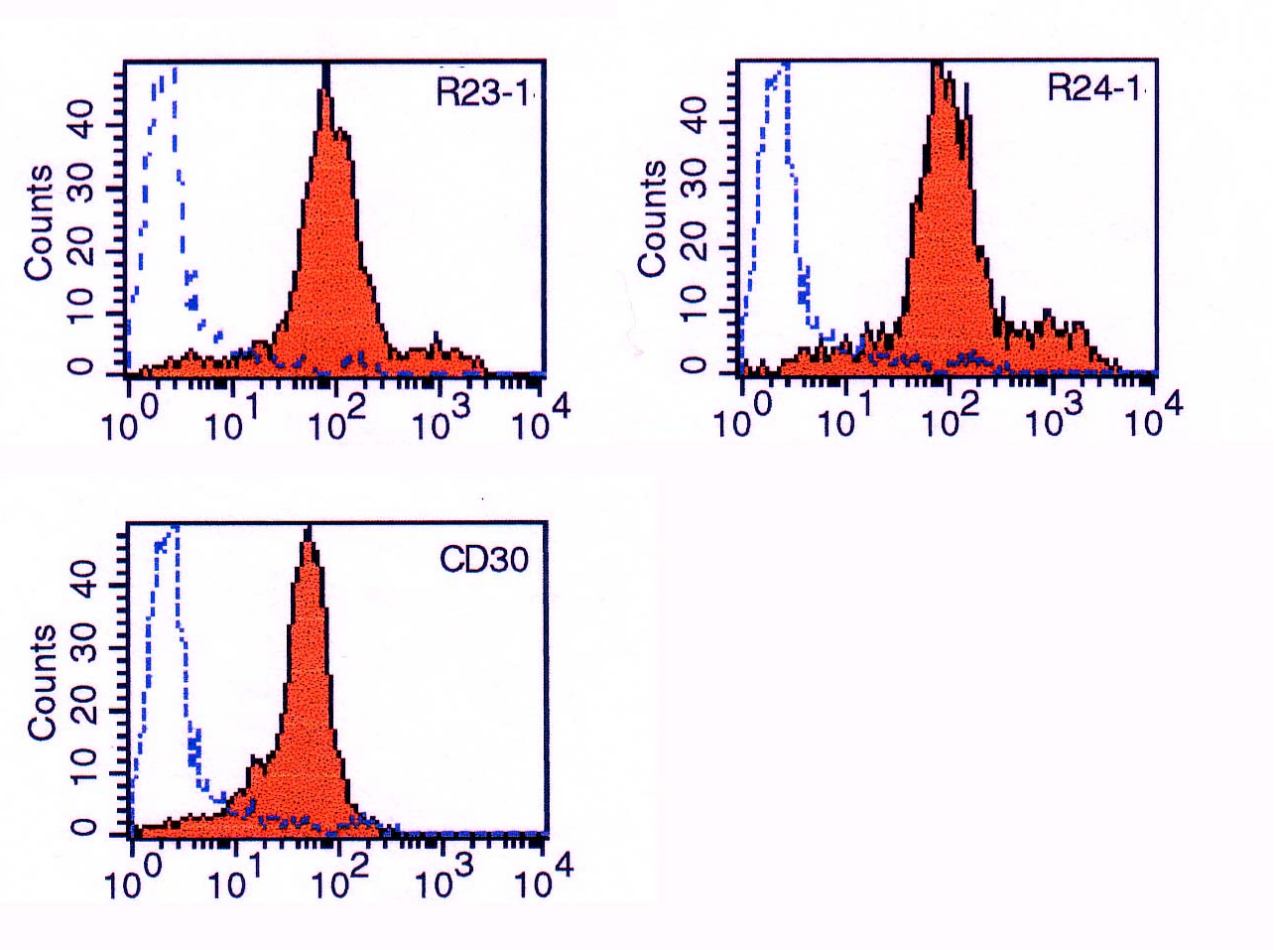
FACS analysis of Hodgkin cell line KMH2 after labeling with clone R23.1, clone R24.1, or CD30 mabs. Blue lines indicate isotype control binding. Solid red curves indicate mab binding.

The two antibodies were reactive against cell surface antigens of almost all CD30+ cell lines as assessed by FACS analysis. Of 14 CD30+ cell lines, R23.1 and R24.1 labelled 12 (86%) (Table 1). Of 14 CD30 negative cell lines, none was labelled by either R23.1 or R24.1 (Table 2). Examples of cell surface labelling of HL cell line KMH2 by the two antibodies as well as anti-CD30 antibody BerH2 are shown in Figure 1. Relative antigen densities as indicated by the position of the fluorescence peak channel tended to vary with each antibody as well as each cell line (data not shown).

**Table 1:** Reactivity with CD30+ cell lines

Cell Line	Isotype Control	CD30	R23.1	R24.1
KMH2 (Hodgkin lymphoma)	-	+	+	+
HDLM (Hodgkin lymphoma)	-	+	+	+
L428 (Hodgkin lymphoma)	-	+	+	+
JB (Anaplastic large cell lymphoma)	-	+	+	+
DEL (Anaplastic large cell lymphoma)	-	+	+	+
SR786 (Anaplastic large cell lymphoma)	-	+	+	+
K562 (Erythroleukemia)	-	+	+	+
HPB/ALL (T lymphoblastic lymphoma)	-	+	+	+
OCI Ly1 (Large B cell lymphoma)	-	+	-	-
OCI Ly3 (Large B cell lymphoma)	-	+	+	+
OCI Ly19 (Large B cell lymphoma)	-	+	-	-
OCI Ly12 (Peripheral T cell lymphoma)	-	+	+	+
OCI Ly17 (Peripheral T cell lymphoma)	-	+	+	+

**Table 2:** Reactivity with CD30 negative cell lines

Cell Line	Isotype Control	BerH2	R23.1	R24.1
U937	-	-	-	-
Jurkat (T acute lymphoblastic leukemia	-	-	-	-
HL60	-	-	-	-
Raji (Burkitt lymphoma)	-	-	-	-
Daudi (Burkitt lymphoma)	-	-	-	-
OCI Ly7 (Large B cell lymphoma)	-	-	-	-
OCI Ly18 (Large B cell lymphoma)	-	-	-	-
OCI M2 (Erythroleukemia)	-	-	-	-
AML-2 (Acute myeloid leukemia)	-	-	-	-
AML-4 (Acute myeloid leukemia)	-	-	-	-
OCI Ly13.1 (Peripheral T cell lymphoma)	-	-	-	-
OCI Ly13.2 (Peripheral T cell lymphoma)	-	-	-	-
OCI Ly8 (B Immunoblastic lymphoma)	-	-	-	-
OCI Ly2 (Large B cell lymphoma)	-	-	-	-

**Figure 2:**
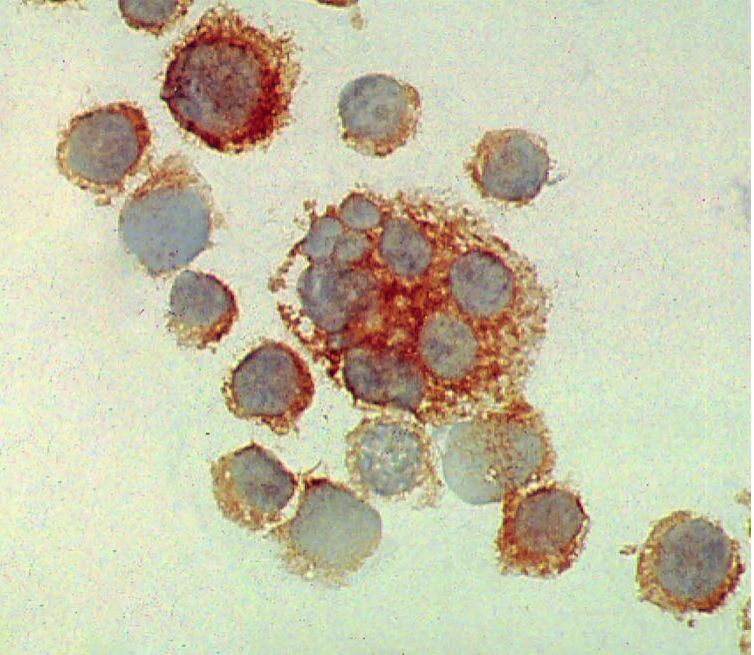
KMH2 cells labelled with clone R24.1 mab. Both membrane and cytoplasmic staining was observed. Magnification ×400.

Both antibodies labelled cytoplasmic antigens in all Hodgkin and ALCL cell lines tested (Table 3). Examples are shown in Figure 2. The pattern was generally diffuse in both mononuclear and multinucleated forms of the cells, though strong staining was observed on the cell membrane. The staining pattern was similar to that observed with the BerH2 anti-CD30 antibody.

**Table 3:** Cytoplasmic immunostaining in Hodgkin lymphoma and anaplastic large celllymphoma cell lines

Cell Line	Isotype Control	Ber-H2	R24.1	R23.1
KMH2 (Hodgkin lymphoma)	-	+	+	+
L428 (Hodgkin lymphoma)	-	+	+	+
HDLM-2 (Hodgkin lymphoma)	-	+	+	+
DEL (Anaplastic large cell lymphoma)	-	+	+	+
JB (Anaplastic large cell lymphoma)	-	+	+	+
SR786 (Anaplastic large cell lymphoma)	-	+	+	+

### Immunohistochemistry in tissue sections

**Figure 3:**
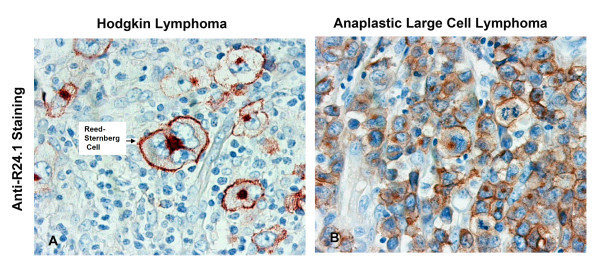
Tissue sections from clinical Biopsies labelled with clone R24.1 mab. On the left panel, a HL tissue section shows intense staining with a membrane and Golgi pattern. On the right panel, an ALCL tissue section shows intense staining of neoplastic ALCL cells. Magnification ×400.

When staining was performed using cryostat sections, both antibodies labelled HRS cells in classical HL cases, of both nodular sclerosis and mixed cellularity subtypes. In lymphocyte predominance (LPHD) cases, one of two cases contained L&H variants which were labelled by both antibodies (data not shown). In formalin fixed sections clone R23.1 mab was not reactive with any cells. In a series of formalin-fixed HL and NHL cases, clone R24.1 mab labelled none of LPHD cases, 100% of classical Hodgkin lymphoma cases, 1 of 4 T cell-rich B cell lymphomas (TCRBCL), 63% of diffuse large B cell lymphomas (DLBCL), 100% of Anaplastic large cell lymphomas (ALCL), and 80% of peripheral T cell lymphomas (PTCL), respectively (Table 4). A survey of non-lymphoid tissue, both benign and malignant (n = 75), including skin melanomas, lung, colorectal, urinary bladder, and mesenchymal tumours revealed no reactivity to the clone R24.1 mab. Examples of positive staining in HL and ALCL clinical biopsies are shown in Figure 3.

**Table 4:** Expression of antigens by neoplastic cells in paraffin sections of clinical biopsysamples

Type	Number	CD30 (% Positive)	R24.1(% Positive)
NLPHD	5	0 (0)	0 (0)
NSHD	21	21 (100)	21 (100)
MCHD	10	10 (100)	10 (100)
LDHD	2	2 (100)	2 (100)
TCRBCL	4	0 (0)	1 (25)
DLBCL	8	4 (50)	5 (63)
ALCL	8	8 (100)	8 (100)
PTCL	5	3 (60)	4 (80)
Total	63	48 (76)	51 (81)

NLPHD = Nodular lymphocyte predominance Hodgkin's disease; NSHD = Nodular sclerosis Hodgkin's disease; MCHD = Mixed cellularity Hodgkin's disease; LDHD = Lymphocyte depletion Hodgkin's disease; TCRBCL = T cell rich B cell lymphoma; DLBCL = Diffuse large B cell lymphoma; ALCL = Anaplastic large cell lymphoma; PTCL = Peripheral T cell lymphoma

### Activation of normal peripheral blood T lymphocytes

**Figure 4:**
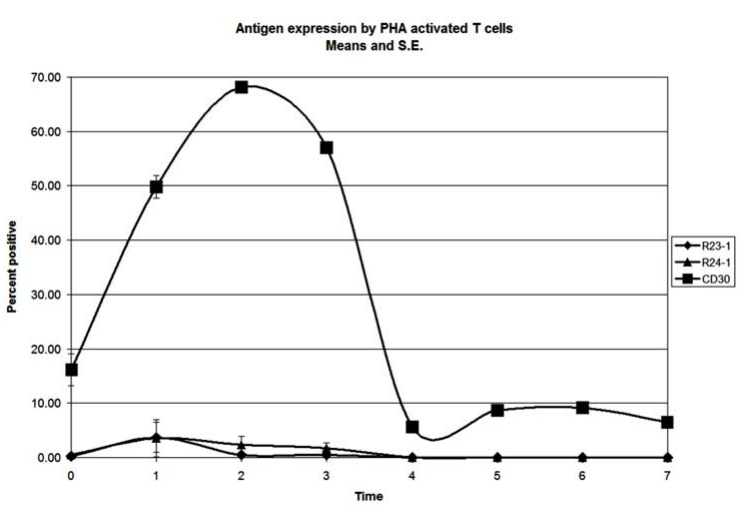
PHA stimulation of T cells. T cells exposed to PHA expressed CD30 within 24 hours, peaking at 48 hours. Expression of R23.1 and R24.1 remained at control levels even up to 7 days after PHA stimulation, indicating that activation of T cells does not induce expression of R23.1 and R24.1 antigens, unlike CD30.

PHA activation of peripheral blood T cells resulted in the expression of CD30 which peaked within 48 hours. CD30 were expressed by 50% of CD3+ T cells within 1 day post simulation, peaking within 48 hours at which time point 70% of CD3+ T cells were CD30 positive, while the antigens recognized by clone R24.1 mab and clone R23.1 mab were not expressed by PHA activated CD3+ T cells even up to 7 days post-stimulation (Figure 4). This indicates that clone R24.1 and clone R23.1 mabs recognize epitopes/antigens unrelated to CD30.

### Competitive binding assays

**Figure 5:**
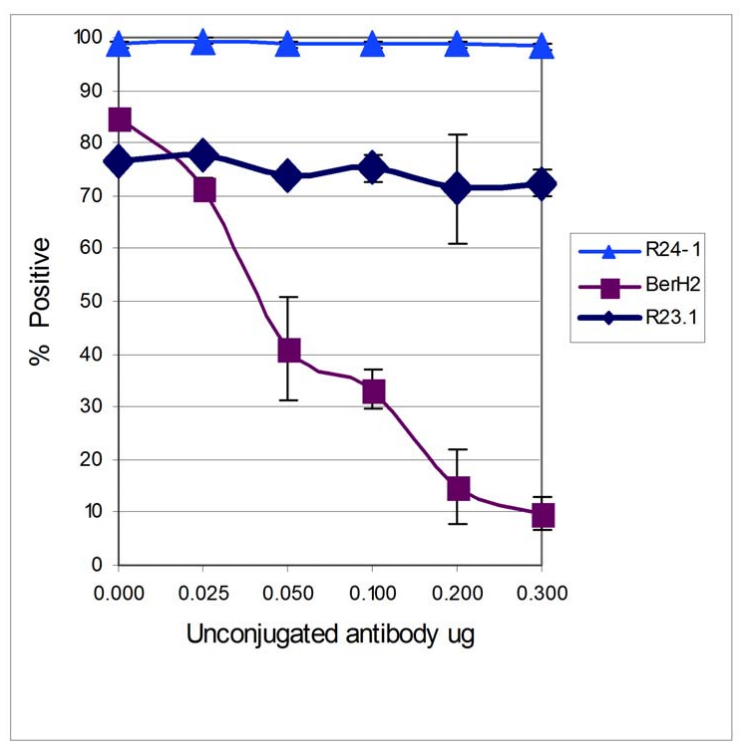
Competitive binding assay. Clone R24.1 and clone R23.1 mabs did not block the binding of anti-CD30 (BerH2 antibody) to KMH2 cells.

Neither clone R24.1 mab nor clone anti-R23.1 mab blocked the ability of three different anti-CD30 antibodies specific for cluster regions A, B, or C of the CD30 molecule, to bind to KMH2 cells (Figure 5). Similarly, none of the anti-CD30 antibodies blocked the binding of clone R23.1 mab or clone R24.1 mab to KMH2 cells (data not shown).

### Western blot analysis and immunoprecipitation

**Figure 6:**
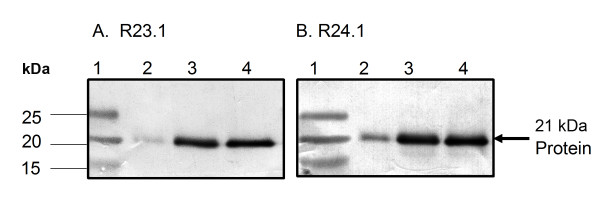
Western-blot analysis of DEL, KMH2 and L-428 cell lysates. A protein with a mass of 21 KDa was detected by clone R23.1 (A) and clone R24.1 (B) mabs in the cell lysates. Lane 1 contains molecular weights and lanes 2–4 contain cell lysates of DEL, KMH2 and L-428 respectively.

**Figure 7:**
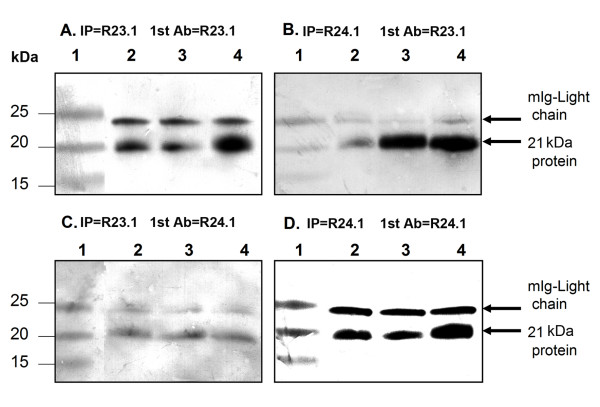
Immunoprecipitation and Western-blot analysis of DEL, KMH2 and L-428 cell lysates. 23.1 (Panel A and C) and R24.1 (Panel B and D). Samples were separated on 4–20% Life-gel and transferred onto nitrocellulose membranes. The membranes were then blocked in 5% non-fat dry milk and probed with monoclonal antibody clones R23.1 (Panel A and B) and R24.1 (Panel C and D) followed by goat-anti-mouse IgG-AP. The membranes were developed in BCIP/NBT. A protein band of ~21 KDa as well as IgG light chain were detected in all three lysates examined. Lane 1 contains molecular weights and lanes 2–4 contain Immunoprecipitated samples of DEL, KMH2 and L-428.

Western-blot analysis of cell lysates indicated that DEL (ALCL), KMH2 (HL) and L-428 (HL) cells expressed a 21 kDa protein which was recognized by clone R23.1 mab and clone R24.1 mab. The level of the 21 kDa protein was lower in DEL than in KMH2 and L-428 cells (Figure 6). The 21 kDa protein was immunoprecipitated by both clone R23.1 and clone R24.1 mabs. Reciprocal probing of the precipitated proteins by Western blot analysis indicated that the clone R24.1 mab reacted with the protein precipitated by the clone R23.1 mab and *vice versa*. These results indicate that clone R23.1 and clone R24.1 mabs reacted with the same 21 kDa protein in DEL, KMH2 and L-428 cells (Figure 7).

## Discussion

CD30, a 120 kDa surface phosphorylated glycoprotein [10], is a marker of activation of T and B lymphocytes [11-15] but because of its constitutive overexpression in classical Hodgkin lymphoma, it is widely used for diagnostic purposes [1,16-18]. It is well known that CD30 expression is not unique to Hodgkin lymphoma, as it is expressed in Anaplastic Large Cell Lymphoma (ALCL)[19], mediastinal large B cell lymphoma [6,20], a subset of nodal diffuse large cell lymphoma[21], large cells in follicular lymphoma [22], nodal and cutaneous diffuse large B cell lymphoma [23-28], peripheral T cell lymphoma [29-32], embryonal carcinoma and other non-lymphoid cells and neoplasms [9,33-37].

With the intention of developing additional antibodies with specificity for non-activation markers of Hodgkin lymphoma, we have characterized 2 novel antibodies that detect a protein constitutively overexpressed in Hodgkin lymphoma. One of the two antibodies, clone R24.1 mab, recognises a formalin-resistant epitope. Although the specificity of the 2 antibodies initially raised the possibility that they are directed against CD30, subsequent experiments proved otherwise. Two B cell lymphoma cell lines (OCI Ly1 and OCI Ly19, Table 1) which are CD30+ lack both R23.1 and R24.1 antigen expression. Unlike CD30, R24.1 antigen expression is not seen in non-lymphoid malignancies, and differences of expression are noted in CD30 negative T cell rich B cell lymphomas, diffuse large B cell lymphomas and peripheral T cell lymphomas, indicating heterogeneity of expression (Table 4). The R23.1 and R24.1 antigen expression is not activation-induced, unlike CD30. Whereas CD30 surface expression by normal T cells peaked within 48 hours after PHA stimulation, neither R23.1 antigen nor R24.1 antigen was expressed on the cell surface by PHA-stimulated T cells. Clone R23.1 and clone R24.1 mabs did not compete with any of the anti-CD30 antibodies directed against the A, B and C extracellular domains of CD30. Western blots and immunoprecipitation studies established that clone R23.1 and clone R24.1 mabs recognize a 21 kDa molecule, which is much smaller than the 120 kDa phosphorylated glycoprotein CD30 [2]. We are in the process of determining the identity of the 21 kDa protein by mass spectrometry and its encoding gene by expression cloning.

A non-activation induced cell surface protein expressed in Hodgkin lymphoma and aggressive non-Hodgkin lymphomas may have immunotherapeutic potential as antibodies that target such a molecule are less likely to be toxic to normal activated immune cells. Anti-CD30 immunotherapy is being explored in pre-clinical models and a few human clinical trials have been conducted, but toxicity has been a problem [38-52] and the long-term sequelae are not yet known. Novel immunotherapy targets and antibodies still require exploration. We are currently evaluating the cytotoxic effects of the two antibodies against HL cell lines in vitro and in vivo HL xenograft models of Hodgkin lymphoma. Tissue microarrays are being assembled from large numbers of Hodgkin lymphoma, diffuse large T and B cell lymphoma, and Anaplastic Large Cell Lymphoma clinical specimens from patients with long-term follow-up in order to determine whether the 21 kDa protein expression has any prognostic significance.

## Conclusion

Clone R23.1 and clone R24.1 mabs recognize a novel 21 kDa cell surface and cytoplasmic protein in HL and ALCL cells as indicated by immunoprecipitation and Western-blot analysis. Clone 24.1 recognizes a formalin-resistant epitope and labels HL, ALCL, and other aggressive forms of NHL. The epitopes R23.1 and R24.1 may potentially be used as a therapeutic target for HL, ALCL and other subsets of NHL. The clone R24.1 mab could be used in patient-selection for such therapies.

## Methods

Animal care and experiments were carried out in accordance with the guidelines of the Canadian Council on Animal Care and all protocols were approved by the Animal Care Committee of the University of Toronto.

Research (immunohistochemistry) was carried out on archived paraffin blocks from clinical biopsies. The use of the archived paraffin tissue was approved by the University of British Columbia Clinical Research Ethics Board in compliance with the Helsinki Declaration (Approval Certificate number H06-60016).

### Preparation of anti-KM-H2 monoclonal antibodies

Animal care and experiments were carried out in accordance with the guidelines of the Canadian Council on Animal Care and all protocols were approved by the Animal Care Committee.

BALB/c mice were immunized with 2 × 10^8 ^live KMH2 B3 cells (a sub-clone of Hodgkin lymphoma cell line KM-H2), by intraperitoneal injection. Fourteen days later, the mice received intraperitoneal injections of 4 × 10^7 ^live KM-H2 B3 cells. On day 24, 100 μL of blood was collected from each mouse and the serum was screened for anti-KM-H2 antibodies by FACS analysis (FACSort; Becton Dickinson, Mississauga, ON, Canada). Sera from all five mice contained antibodies that bound to KMH2 cells, whereas sera from unimmunized control mice had no detectable anti-KMH2 antibodies. On day 33, the mice were given intraperitoneal injections of 10^6 ^live KMH2 cells. Serum was collected and screened for anti KMH2 binding activity by FACS analysis. The mouse with the highest titer of KMH2 binding activity was boosted by intravenous injection of 10^6 ^live KM-H2 cells on day 91. Five days later, the mouse was sacrificed humanely and the spleen was harvested aseptically. Spleen cells were fused with hypoxanthine guanine phosphoribosyl transferase (HGPRT) deficient P3X63-AG8.653 [53] mouse myeloma cells (non-secretor) in a 5:1 ratio of spleen cells to myeloma cells. Fused cells were selected in hypoxanthine, aminopterin and thymidine (HAT) medium using a final working concentration as follows: 100 μM hypoxanthine, 0.4 μM aminopterin, 16 μM thymidine (Sigma-Aldrich Canada Ltd.; Oakville, ON, Canada) [53]. Hybridoma supernatants were screened for IgG and IgM production by ELISA. Six IgM and 10 IgG producers were identified. Supernatants from the 10 IgG producers were screened by FACS analysis for anti-KMH2 antibodies and 5 of the 10 were found to be producing such antibodies. These were cloned twice by limiting dilution and the supernatants were re-screened by FACS for anti-KM-H2 activity. Two clones were selected for further analysis (R23.1 and R24.1).

### Cell lines and cell culture

Cell lines were grown in Iscove's Modified Dulbecco's Medium (IMDM) supplemented with penicillin and streptomycin, 1% Fungizone (Gibco/BRL, Gaithersburg, MD, USA), and 10% fetal bovine serum (FBS) (Invitrogen, Burlington, ON, Canada). Hodgkin lymphoma (HL) cell lines, KMH2 and L-428, and anaplastic large cell lymphoma (ALCL) cell line Del were purchased from DSMZ (Braunschweig, Germany). The cell lines HPB/ALL (T-cell lymphoblastic non-Hodgkin's Lymphoma), K562 (Erythroleukemia), U937 (Monocytic leukemia), HL60 (promyelocytic leukemia), DAUDI (Burkitt's lymphoma), MOLT-4 (T-cell Lymphoblastic leukemia), and Jurkat (Acute T-cell leukemia) were purchased from the American Type Culture Collection (ATCC). OCI Ly1, Ly2, Ly3, Ly7, Ly18, Ly19 (Large B-cell Non-Hodgkin's Lymphomas); OCI Ly8 (B-immunoblastic lymphoma); OCI Ly12, Ly13.1, Ly13.2 and Ly17 (T-cell Non-Hodgkin's Lymphomas); OCI-M2 (Erythroleukemia); AML-2 and AML-4 (Acute Myeloid Leukemia) were all generous gifts from Dr. Hans Messner (Ontario Cancer Institute/Princess Margaret Hospital, University Health Network, Toronto, ON, Canada).

### Determination of cell surface expression of antigens on cell lines

Cells were washed (4 min.; 300 × g) two times in calcium and magnesium-free phosphate buffered saline (PBS^-^) and then treated as follows to block non-specific binding of mabs. Cells undergoing direct staining were blocked with 10 μg of pure mouse IgG (Sigma, Oakville, ON, Canada) for 20 minutes at 4°C. Cells undergoing indirect staining were blocked with 10% normal goat serum (NGS) for 20 minutes at 4°C. Cells were then washed in PBS^-^. Cell concentrations were adjusted so each tube received 1 × 10^6 ^cells. CD30-FITC (Dako, Mississauga, ON, Canada) was added at 1 μg per tube, the two mabs were added at 1 μg per tube, the mouse IgG1 isotype controls (IgG1-FITC and mouse IgG unconjugated) were both added at 1 μg per tube. Primary antibody staining was done for 30 minutes at 4°C in the dark. Cells were then washed in PBS^-^, and goat anti-mouse IgG -PE (Dako) was added and allowed to incubate for 30 minutes at 4°C in the dark. Viability was assayed by propidium iodide exclusion. Cells were washed in PBS^- ^and 300 μl of fixative (1% paraformaldehyde) was added. Cells were analyzed on a FACSort flow cytometer (Becton Dickinson) within 15 minutes.

### Immunocytochemistry and immunohistochemistry

Immunocytochemistry was performed using monoclonal antibody BerH2 (Dako), and the 3 monoclonal antibodies. Cytospins were prepared using the cell lines KMH2, L428, HDLM-2, JB, DEL, and SR786, as follows. Cells were washed 1× PBS^- ^and centrifuged for 4 min. at 300 × g. Cells were then added to a 20% FBS/PBS^- ^solution and 25 μl aliquots of this solution was added to 100 μl of PBS^- ^and spun for 5 min. at 50 × g (Cytospin 2; Shandon). Slides were allowed to air dry overnight.

Identical sets of slides were fixed in 2% paraformaldehyde at room temperature (RT) or cold acetone (4°C) for 10 minutes. Slides were then washed in PBS^-^. Slides were blocked in 10% normal goat serum (NGS) diluted in antibody dilution buffer (Dako) for 20 min. at RT. The slides were then washed in PBS and stained with the primary antibody for one hour (diluted in antibody dilution buffer as above) in the following concentrations: R23.1 and R24.1, at 16 μg/ml; CD30 at 8 μg/ml; Isotype control at 16 μg/ml (in antibody dilution buffer). Slides were washed in PBS and incubated with 1/200 dilution of biotinylated goat anti-mouse IgG (Zymed, South San Francisco, CA, USA) for 20 minutes, washed in PBS, and then stained with 1/4 dilution of streptavidin-biotin complex labelled with horseradish peroxidase for 20 min. (Ultra Streptavidin Detection System; Signet). The enzyme reaction was developed with AEC (3-amino-9-ethyl carbazole) and counterstained with hematoxylin.

Immunohistochemistry was carried out on deparaffinized tissue sections (5 micron thickness). Endogenous peroxidase was quenched by treating the sections with 3% hydrogen peroxide for 10 minutes, followed by rinsing in PBS. Antigen retrieval was done by pepsin digestion (0.4% pepsin for 3 minutes, then rinsing in PBS^-^) prior to labelling. Non-specific binding was blocked with 10% human AB serum for 20 minutes at room temperature. R24.1 hybridoma supernatant was used at 1/4 dilution whereas purified R24.1 was used at 16 μg/ml. The slides were incubated at room temperature for 60 minutes, rinsed in PBS and then incubated with biotinylated goat anti mouse IgG (Zymed) at 1/200 at room temperature for 20 minutes, followed by rinsing in PBS. They were then incubated for 20 minutes in 1/4 dilution of streptavidin-biotin complex labelled with horseradish peroxidase for 20 min. (Ultra Streptavidin Detection System; Signet). After rinsing in PBS^-^, the slides were incubated with AEC for 10 to 20 minutes, rinsed in PBS^- ^and counterstained with Mayer's hematoxylin.

### Chemicals and reagents

IMDM media was purchased from StemCell Technologies Inc. (Vancouver, Canada). Protein G-sepharose beads were purchased from Sigma (St. Louis, Mo, USA). Fungizone was purchased from Gibco (Grand Island, NY, USA). Proteinase inhibitor cocktail, 30% polyacrylamide (acrylamide: bis = 29:1) and substrate BCIP/NBT were purchased from Sigma (St. Louis, MO, USA). Seize Classic Mammalian immunoprecipitation Kit was purchased from Pierce biotechnology (Rockford, IL, USA).

### Other antibodies

Monoclonal Anti-CD30 was purchased from BD Pharmingen (San Diego, CA, USA). Secondary antibody, goat anti-mouse IgG Alkaline phosphatase, was purchased from Sigma (St. Louis, MO, USA).

### Lysate preparation

Lysis buffer (RIPA) [54] was added to the cell pellets at 10:1 (V/W) ratio and the samples were then incubated on ice for 10 minutes. The debris was removed by centrifuging at 10000 g for 10 min. Proteinase inhibitor cocktails (Sigma, MO, USA) were added to lysis buffer to reduce the degradation.

### SDS-PAGE and Western-blot analysis

The protein samples were analysed on 4–20% gradient gel (Life Gels, Life Therapeutics, Clarkston, GA). The gels were run at 140 V for 70 min. After the proteins were resolved by SDS-PAGE, the gels were incubated in 1 × transfer buffer [54] for 30–60 min. Separated proteins were transferred onto nitrocellulose membrane (Millipore, Billerica, MA, USA) with semi-dry-Transfer apparatus (BioRad, Hercules, CA, USA). After the nitro-cellulose membranes were blocked in 2% BSA-PBS (pH 7.2) for 2 hours at room temperature, they were probed with primary and secondary antibodies sequentially at 4°C overnight and for 2 hrs at room temperature followed by 4 washes in PBS. The primary antibodies were diluted clone R23.1 mab, clone R24.1 mab and anti-CD30, and the secondary antibody was goat anti-mouse IgG alkaline phosphatase. The protein bands were finally visualized in BCIP/NBT substrate (Sigma, MO, USA).

### Immunoprecipitation

Immunoprecipitation was performed using protein-A beads from Sigma (St. Louis, MO, USA). Briefly, 400 μl of cell lysates were precleared for 2 hrs at 4°C with 100 μl of 50% protein-A beads absorbed with normal mouse IgG. The precleared lysates were then incubated overnight with 100 μl of protein-A-antibody beads at 4°C. The beads were centrifuged at 10,000 g for 1 min and washed 4 times with 1.5 ml of PBS (pH 7.2). The washed beads were boiled for 10 min in 100 μl of SDS-sample buffer and the samples were analyzed by SDS-PAGE and Western-blot analysis.

Alternatively, the samples were eluted from the beads in 8 × 400 μl of glycine (0.01 M, pH2.8). The eluted samples were pooled and neutralized with 1 M Tris (pH7.4) and concentrated with Microcon YM-10 (Millipore, Billerica, MA, USA).

### Activation of normal peripheral blood T lymphocytes

Peripheral blood from healthy volunteers, who provided written informed consent, was obtained by venipuncture. Lymphocytes were isolated by density gradient centrifugation using Ficoll-Paque (Pharmacia Biotech, Uppsala, Sweden), washed twice in PBS^- ^and then cultured in IMDM supplemented with penicillin, streptomycin, 1% Fungizone (Gibco/BRL), 10% FBS and 100 μg/ml of phytohemagglutinin-M (PHA-M). Cells were analyzed at 0, 24, 48, 72, 96, 120, 144, and 168 hours after initiation of culture. Analysis was based on viable (Propidium iodide negative) CD3+ cells co-expressing CD30, R23.1, or R24.1.

### Competitive binding assays

KMH2 cells were harvested and washed twice in PBS^- ^(300 × g; 4 min.). Cells were blocked in 20 μg/ml of total mouse IgG (Sigma; I-5381) for 45 min. at 4°C. Cells were then washed twice in PBS^- ^(300 × g; 4 min.) and resuspended in PBS^-^. Antibodies were added in a cocktail approach (unconjugated plus conjugated) to tubes (12 × 75 mm, 5 ml polystyrene; Falcon 35-2008). Unconjugated antibodies were added to a fixed (0.1 μg/ml) conjugated amount. Varied amounts (0 to 0.3 μg/ml) of unconjugated anti-CD30 antibody BerH2 (Dako), R23.1, or R24.1 were mixed with fixed amounts of FITC-conjugated anti-CD30 antibodies BerH2 (Dako; M0751), phycoerythrin conjugated AC10 (Ancell; 179-020), FITC-conjugated R23.1, or R24.1. Ki-1 was not available as a conjugated antibody, thus CD30 clone Ki-1 unconjugated (Immunotech) was mixed with fixed amounts (0.1 μg/ml) of FITC-conjugated R23.1 or R24.1 antibodies. Mouse IgG-FITC/or R-PE (Immunotech) was used as isotype control as relevant. One × 10^5 ^cells were added to each tube, mixed gently, and allowed to stain for 25 min. at 4°C. Labelled cells were washed once, incubated with 0.5 μg of propidium iodide (Pharmingen), washed, and then fixed in 1% paraformaldehyde and analyzed on a FACSort (Becton Dickinson). Cells were gated for viable cells by excluding those positive for propidium iodide.

## Competing interests

The author(s) declare that they have no competing interests.

## Authors' contributions

All authors have read and approved the final version of the manuscript. MZ participated in the design of the study, performed immunoprecipitation and Western-blot analysis. FF contributed with cell cultures, scientific discussion and manuscript preparation. JBC participated in the hybridoma establishment and performed cell proliferation and other in vitro assays. BS and RDG contributed with data interpretation, scoring of immunohistochemistry and manuscript discussion. DB was the principal investigator and director of the laboratory and responsible for the design of the study and its coordination. He interpreted the results independently of the author 1, and contributed in the preparation of the manuscript.
